# Point-of-care testing of plasma free hemoglobin and hematocrit for mechanical circulatory support

**DOI:** 10.1038/s41598-021-83327-5

**Published:** 2021-02-15

**Authors:** Dong Ah Shin, Jung Chan Lee, Heean Shin, Young-Jae Cho, Hee Chan Kim

**Affiliations:** 1 Interdisciplinary Program in Bioengineering, Graduate School, Seoul National University, Seoul, 08826 Republic of Korea; 2 Department of Biomedical Engineering, College of Medicine and Institute of Medical and Biological Engineering, Medical Research Center, Seoul National University, Seoul, 03080 Republic of Korea; 3 Institute of BioEngineering, Seoul National University, Seoul, 08826 Republic of Korea; 4 Division of Pulmonary and Critical Care Medicine, Department of Internal Medicine, Seoul National University College of Medicine, Seoul National University Bundang Hospital, Seongnam, 13620 Republic of Korea

**Keywords:** Diagnostic markers, Lab-on-a-chip, Biomedical engineering

## Abstract

Hematological analysis is essential for patients who are supported by a mechanical circulatory support (MCS). The laboratory methods used to analyze blood components are conventional and accurate, but they require a mandatory turn-around-time for laboratory results, and because of toxic substances, can also be hazardous to analysis workers. Here, a simple and rapid point-of-care device is developed for the measurement of plasma free hemoglobin (PFHb) and hematocrit (Hct), based on colorimetry. The device consists of camera module, minimized centrifuge system, and the custom software that includes the motor control algorithm for the centrifuge system, and the image processing algorithm for measuring the color components of blood from the images. We show that our device measured PFHb with a detection limit of 0.75 mg/dL in the range of (0–100) mg/dL, and Hct with a detection limit of 2.14% in the range of (20–50)%. Our device had a high correlation with the measurement method generally used in clinical laboratories (PFHb R = 0.999, Hct R = 0.739), and the quantitative analysis resulted in precision of 1.44 mg/dL for PFHb value of 14.5 mg/dL, 1.36 mg/dL for PFHb value of 53 mg/dL, and 1.24% for Hct 30%. Also, the device can be measured without any pre-processing when compared to the clinical laboratory method, so results can be obtained within 5 min (about an 1 h for the clinical laboratory method). Therefore, we conclude that the device can be used for point-of-care measurement of PFHb and Hct for MCS.

## Introduction

Mechanical circulatory support (MCS) systems refer to the system for artificially circulating a patient’s blood using mechanical devices, such as a blood pump, and typically includes an extracorporeal membrane oxygenator (ECMO), a cardiopulmonary bypass (CPB) device, and a ventricular assist device (VAD)^1,2^. The system consists of a pump, a blood circuit, and a control system for regulating the flow of the blood^2^. However, this MCS system can cause various hemodynamic problems, such as thrombus generation or hemolysis when a large local pressure gradient occurs inside the pump, or when the proper rate is not supplied^3,4^. Hemolysis is a phenomenon in which red blood cells are damaged and hemoglobin is released into plasma, and it can be diagnosed by measuring the hemoglobin dissolved in plasma^3,5,6^. It may occur in patients supported by the MCS system, and result in death of the patient by reducing oxygen carrying capacity^7,8^. Therefore, in order to prevent such problems, hemoglobin levels in the plasma (Plasma free hemoglobin, PFHb) should be periodically monitored; and if hemolysis occurs, the blood circuit should be replaced, or blood transfusion should be quickly performed^9^. Also, commonly used parameters include hematocrit (Hct) and hemoglobin (Hb) levels^8,10^. Hct is an important parameter of disease and dehydration related to total plasma, such as diabetes, venous thrombosis, and peritonitis, which may appear in patients undergoing mechanical circulation^11^. Hb is also an important parameter that can affect the oxygen transport capacity of the patient^8,12,13^. Therefore, periodic monitoring is also essential for these parameters as much as PFHb levels, since abnormal range of Hct and Hb levels can cause fatal effects on patients, such as cardiovascular and endocrine disorders^14,15^.

To measure these parameters of patients supported with mechanical circulation, the blood samples from the patient are sent to the laboratory in the hospital, but a turnaround time to obtain the results can takes approximately 30–90 min. This is because the turnaround time depends on the number of samples, the time the samples arrive in the laboratory, the sample analysis time, and the relative urgency^16,17^. Therefore, it is very difficult to monitor these parameters of the emergency patient who needs the result in a short period of time. Also, the measurement of PFHb and Hb in clinical laboratories requires a pre-analytical process for each assay. PFHb requires centrifugation because it requires the separation of blood cells and plasma before measurement, which takes about 10 min^18^. A common method for Hb measurements is spectroscopy using the cyanide methemoglobin method. This method can be measured at high sensitivity, even in small quantities, but to produce cyanide hemoglobin, chemical substances such as potassium cyanide (KCN) must be used to lysis red blood cells for hemoglobin release and induce cyanide. Since KCN is a toxic chemical that can lead to death even in small quantities, it has the potential to be harmful to the health of the user (the analyst in the laboratory)^18,19^.

Since the parameters PFHb, Hct, and Hb are the most common and necessary for the diagnosis of blood-related diseases, various studies have been carried out on how to measure these parameters^20^. Recently, there has been a study on developing a hemolysis diagnosis device using a mobile phone that measures PFHb level using color difference values^18^. It has the advantage of being able to diagnose on-site and show the results quickly with a mobile phone, but it takes more than 10 min to separate plasma and blood cells. In addition, the measurement results may vary depending on the camera performance of the mobile phone and ambient light. Detection methods for Hb have been developed based on the photothermal response of iron oxide in the Hb^21^. This method can measure Hb using very small amounts of blood without the specific reagents with high accuracy, but it requires the use of an expensive laser module that produces a photothermal effect. In addition, it can be difficult to measure PFHb, because generally the level of the PFHb is lower than the measurement limit (0.12 g/dL) of this method. Since the mentioned studies can only measure a single parameter, they are not suitable for patients supported by the MCS systems.

Therefore in this study, we present a device design and method for analyzing the level of hematological parameters of PFHb, Hct, and Hb that can be applied to the MCS. The device is developed using colorimetric analysis, based on the fact that as the level of PFHb increases, the color of the plasma becomes redder^22,23^. Also, a customized channel cartridge was used to measure all three parameters with a small amount of blood (35 µL), and the centrifuge system was integrated into the device, so that centrifugation and analysis could be performed simultaneously. To verify the performance of the device and its suitability for use in the MCS environment, we performed the device validation with blood samples obtained during ECMO animal experiments.

## Results

### Device for point-of-care measurement

**Figure 1:**
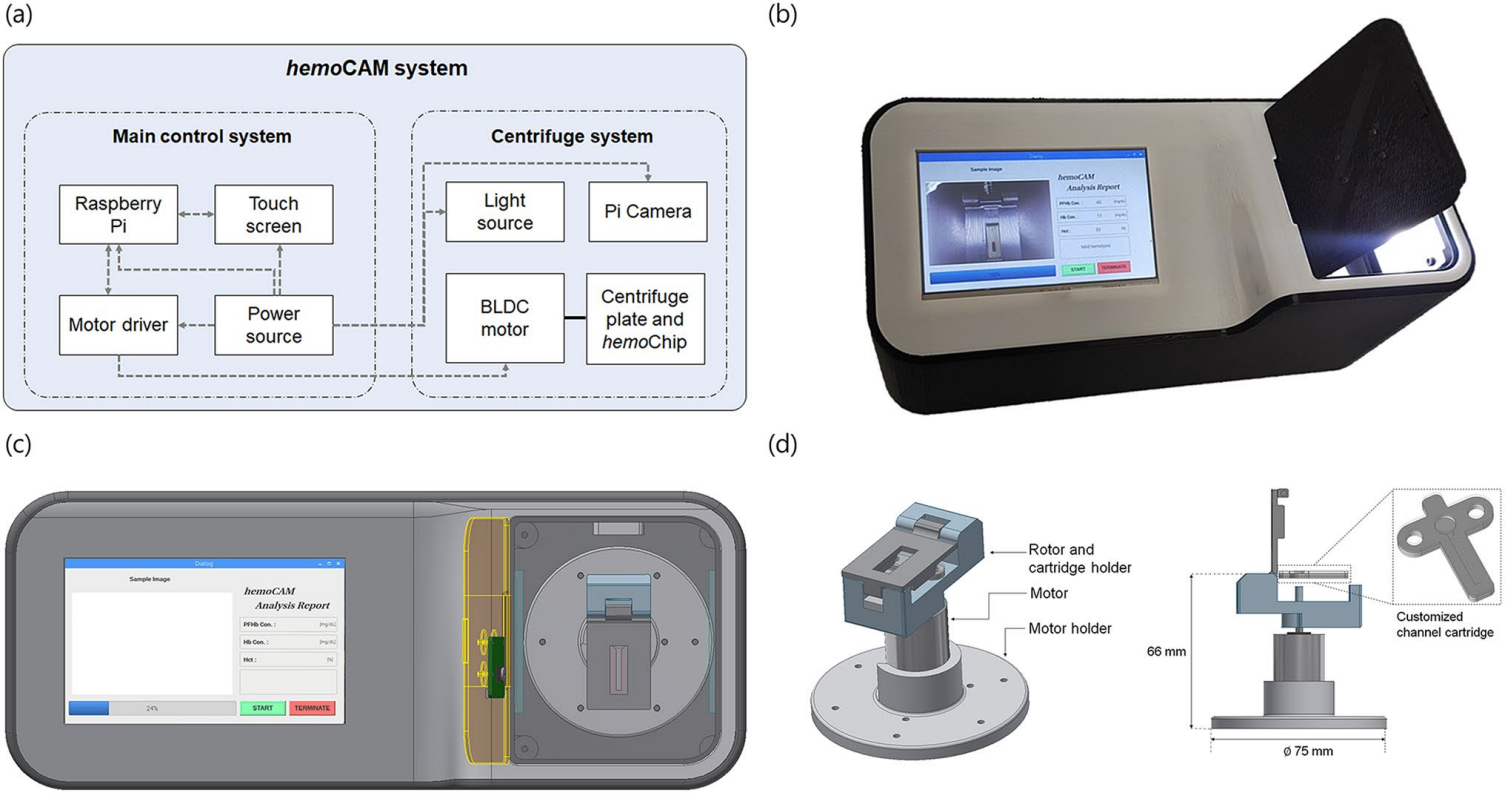
Point-of-care device for hematological analysis. (**a**) Schematic of the device, which is separated into two parts (main control system and centrifuge system). (**b**) The prototype of the device. (**c**) Top view of the device. The left side shows the touch screen of the main control system. The centrifuge system is on the right side, and the camera module is placed on a cover above the centrifuge system to obtain a channel image of the cartridge. (**d**) The centrifuge system consists of motor, motor holder, rotor, and cartridge holder. A customized channel cartridge is fitted to the cartridge holder.

To develop a portable and stand-alone device for use in MCS environments, we designed a device that integrates the centrifuge and the main control part (Fig. 1a and Supplementary Fig S1). The centrifuge part consists of: (1) a BLDC motor, which is the main part of the centrifuge system; (2) a holder to help fix the customized channel cartridge, and to facilitate the acquisition of microchannel images; (3) a light source that provides a constant light intensity inside the device; and (4) a camera module (Pi camera ver.2.1) that is compatible with the Raspberry Pi 3. The main control part of the device consists of (1) a user-friendly touch screen to operate the device, and (2) a Raspberry Pi3 to control the motor, camera module, and image processing^24^. The overall dimensions of the device are 290 mm (L) × 115 mm (W) × 130 mm (H), and 1.1 kg in weight, and the entire housing was fabricated using a 3D printer (Stratasys F123 Series, Stratasys, Israel) (Fig. 1b,c). The channel cartridge is designed for centrifugation and imaging for a small amount of blood (Fig. 1d). To operate the device, first the chip is mounted on the holder, and the user presses the start button on the touch screen, whereupon the centrifugation, acquisition of the image, and the image processing from the camera are sequentially performed by custom software. The software includes a motor control algorithm for centrifugation, and an image processing algorithm for analysis. This integrated system allows the analysis to be performed in a short time, without complicated procedures.

### Image processing algorithm for blood analysis

**Figure 2:**
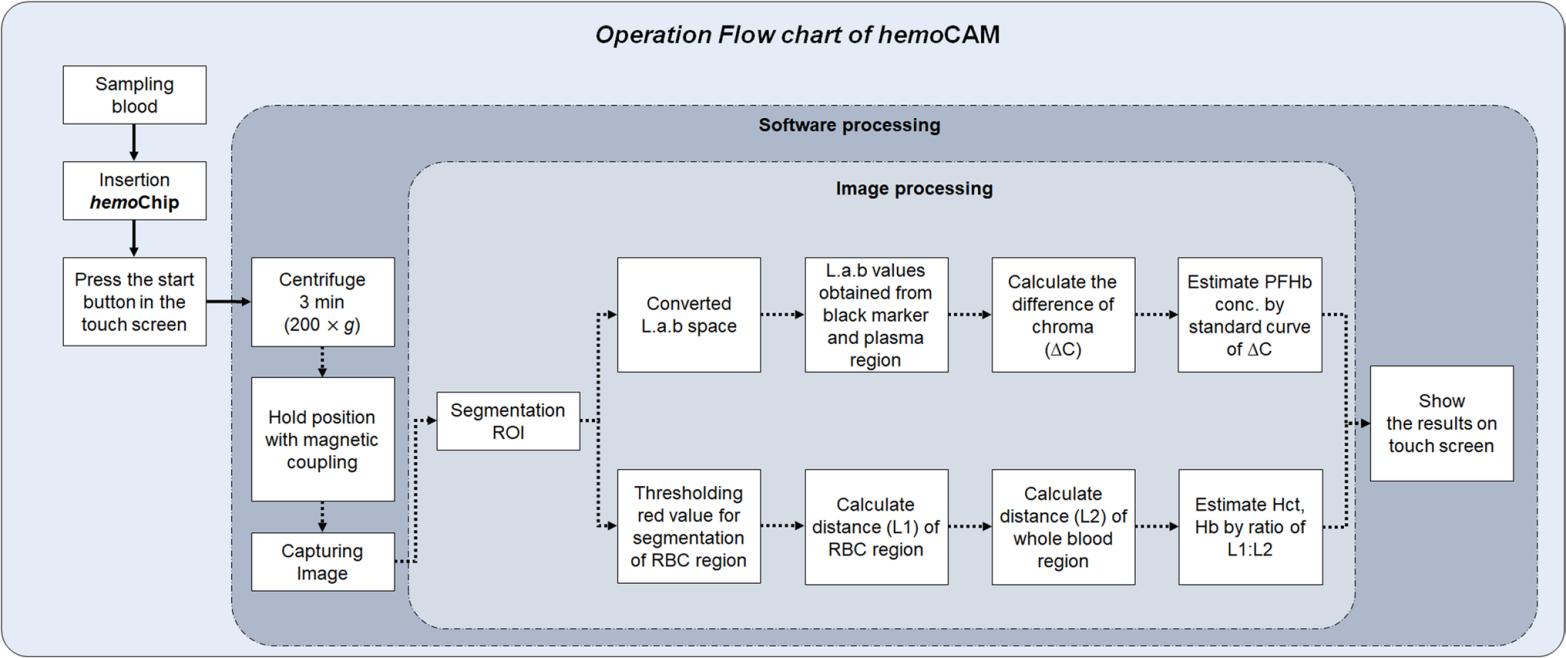
Operation flow chart. After inserting the cartridge, the custom software program starts the centrifugation. At the end of centrifugation, the cartridge holder is stopped by magnetic coupling with the housing at the specified location for image capture. Image analysis is performed simultaneously with the color space analysis of PFHb, Hct, and Hb. The analyzed result is printed on the touchscreen, and all of this process is completed within only 4 min.

Figure 2 shows the operating flowchart of the entire system. The image analysis process begins with the segmentation of the region of interest (ROI) in the acquired image after the centrifugation. Afterwards, the PFHb measurement process and the Hct measurement process are performed simultaneously in the program.

The ROI at the initial step of image processing is segmented to cover the entire channel of the cartridge. Red, green, and blue (RGB) values are extracted for each pixel, and the channel is divided into the region of red blood cells (RBCs) and plasma based on the specific threshold of red color. In order to measure the PFHb level, the second ROI (2nd ROI) with the size of (20 × 100) pixels is segmented from the previously distinguished plasma region. RGB values are extracted from this 2nd ROI, and then converted to CIELab values. The CIELab is a color space defined by the International Commission of Illumination, which makes it possible to closely match the color difference that the human eye can detect, and the color difference expressed in numerical values in the color space^25^. The color components are represented by L *, a *, b *, where, L * is the brightness, a * is the degree of red and green, and b * is the degree of yellow and blue. L.a.b. values were extracted from the region of black marker and the plasma in the 2nd ROI, respectively^18,26^. The chroma difference (△C) was calculated using the a * and b * values of the 2^nd^ ROI, and converted to the PFHb levels by the calibration curve (Eq. 1):

1 $$\Delta \mathrm{C}= \sqrt{{({a}_{m}- {a}_{s})}^{2}+ {({b}_{m}- {b}_{s})}^{2}}$$

where, ${a}_{m}$ and ${b}_{m}$ are the CIELab components of the region of black markers, and ${a}_{s}$ and ${b}_{s}$ are the CIELab components of the region of the plasma in the 2nd ROI. To exclude the change of the color components caused by the brightness, the difference between the average brightness value (L) of the black marker obtained from the calibration images and the brightness value of the black marker obtained from each sample image was added as an offset value to the chroma difference.

To measure the Hct, the coordinate values of the lowest and the highest row in the pixels of the RBCs region are obtained, and the distance between the rows (L1) represents the volume of the RBCs. The distance between the highest row of the plasma region and the lowest row of the red blood cell region is expressed as the length (L2), which represents the total blood volume in the channel of the cartridge. The Hct level is calculated as the ratio of L1 and L2 as in Eq. (2):

2 $$\mathrm{Hct }\left(\mathrm{\%}\right)=\left(\frac{{L}_{1}}{{L}_{2}}\right)*100$$

where, Hct is the percentage of the RBCs volume in the whole blood volume, which is generally the same as three times the levels of the hemoglobin level. Therefore, we used the Hct method to calculate the hemoglobin level (Eq. 3):^27^

3 $$\mathrm{Hct }\left(\mathrm{\%}\right)=3*Hb$$

### Standard curve for the quantification of plasma color

**Figure 3:**
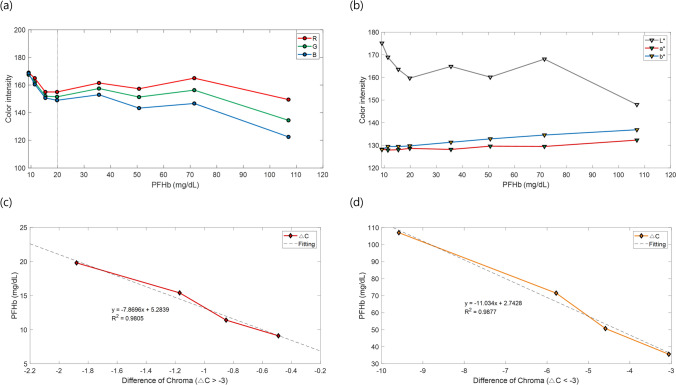
Standard curves of the PFHb levels*.* (**a**) Intensities of the RGB color channels according to PFHb levels. (**b**) Intensities of the L.a.b. color spaces converted from RGB. (**c**,**d**) Standard curves as measured by the proposed device. (**c**) Calibration curve in a region where the saturation difference (∆C) is greater than − 3, and (**d**) calibration curve in a region where the ∆C is less than − 3.

The method to measure the level of PFHb is based on the phenomenon that as the hemolysis becomes more severe, the redness of the color of plasma increases. To obtain a change of the color intensity according to the degree of hemolysis, we induced a severe hemolysis to the blood sample of swine. The stressed blood was centrifuged to collect only the plasma, and we adjusted different levels of the PFHb by diluting the collected plasma to obtain the required range in the clinical setting. Images of each diluted plasma sample were obtained using our device, and a relationship was obtained by comparing the color intensity of the plasma extracted from the image with the PFHb level measured by the actual lab test. Figure 3a shows the change of intensities in RGB channels according to PFHb levels. It shows that the intensity of the red channel is higher than that of the green and blue. Also, Fig. 3b shows the change of intensities in L,a,b color space according to the PFHb levels. The intensity of light is similar to that of the RGB channel, but a * and b * spaces show a different graph of change, compared to the RGB channel. Therefore, because the intensity of the light can be interpreted to have a significant effect on the RGB channel, the chroma difference that reflects only the changes in a* and b* spaces is adopted as a standard curve, to exclude the change of light. As shown in the results, since the gradient change of the color channel does not have linearity, and varies based on the specific level of PHFb (20 mg/dL), different calibration curves were obtained based on this. Figure 3c,d show the results of the PFHb levels according to the chroma differences calculated in the developed device, show two different calibration curves based on the chroma difference value of − 3, and both have R-squared values that are greater than 0.98.

### Validation of the PFHb, Hct, and Hb values

**Figure 4:**
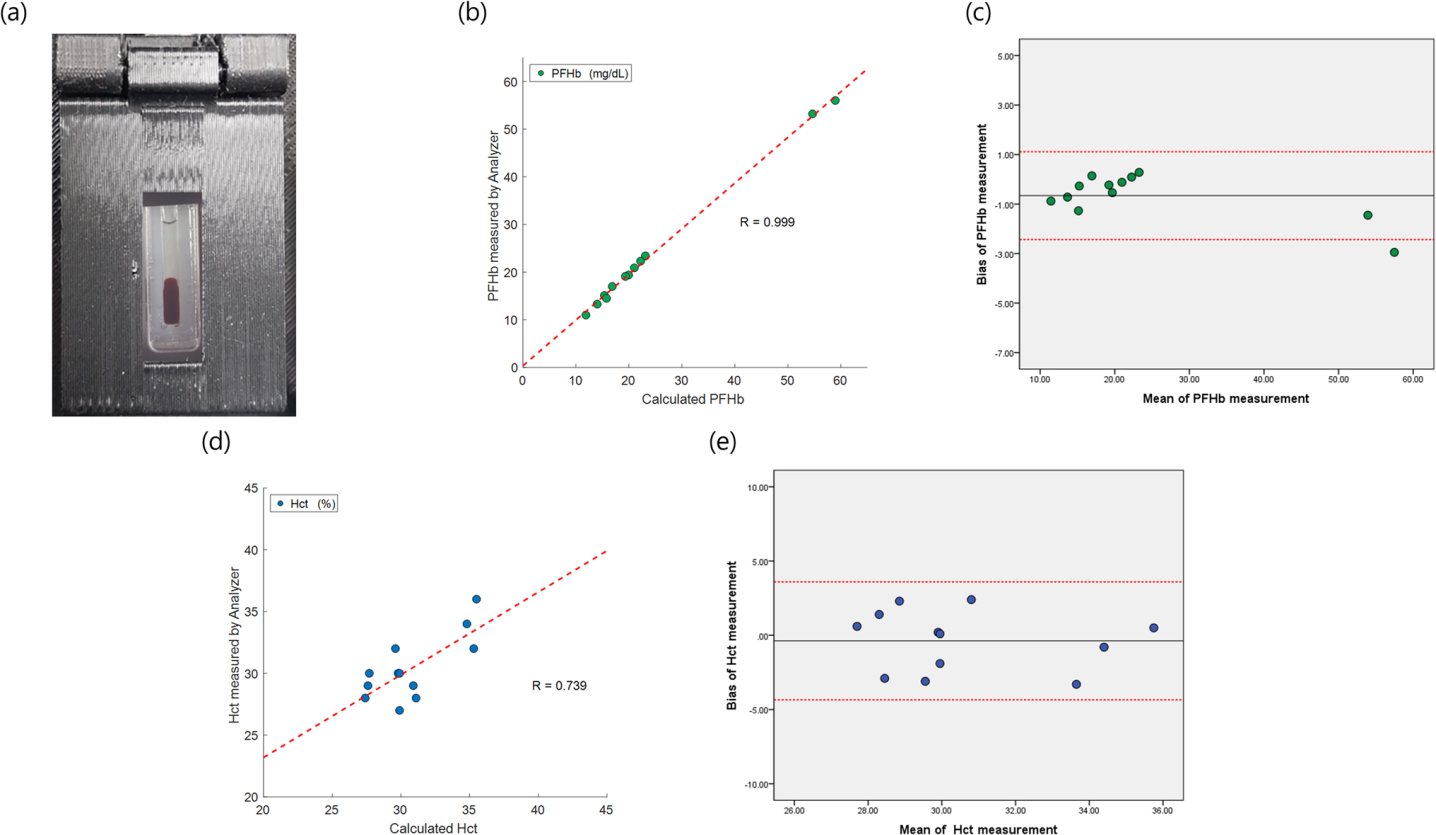
In vitro validation of the hematological analysis during in vivo study of the Venous-arterial extracorporeal membrane oxygenation (VA-ECMO) system using swine model. (**a**) Obtained image from the camera module after centrifugation. (**b**–**e**) Comparison results between our device and lab test results. (**b**) Linear regression analysis of PFHb levels with slope of 0.958, intercept of 0.366, and R of 0.999. (**c**) Bland–Altman analysis of PFHb levels, showing a mean bias of − 0.38 mg/dL and a 95% confidence interval of (− 2.44 to 1.12) mg/dL. (**d**) Linear regression analysis of Hct levels with slope of 0.669, intercept of 9.812, and R of 0.739. (**e**) Bland–Altman analysis of Hct levels, showing a mean bias of − 0.38% and a 95% confidence interval of (− 4.35 to 3.60) %.

To validate the performance of the device in the MCS environment, we performed the evaluation of the device during ECMO animal experiments with the swine model. Blood samples of about 10 mL were obtained through an artery line, and from the sample, the amount 35 µL required for the developed device was extracted, and the rest of the blood was used to get the gold standard level of CBC, PFHb, etc. Figure 4a is an image of the channel cartridge obtained after centrifugation and the analysis algorithm started using this image according to the procedure in Fig. 2.

Blood samples were obtained at one hour intervals during ECMO experiments, and collected blood samples (n = 12) were compared with the developed device and the lab test results. Our device took about 5 min to get the results of the analysis. Also, we found that the results measured by our device were very similar to those of the lab test. Regression analysis and Bland–Altman analysis were performed on the PFHb and Hct to verify the reliability of our device^28^. The results of PFHb measured by our device and lab test correlated well (R value of 0.999, n = 12) (Fig. 4b). In addition, Bland–Altman analysis of the PFHb (Fig. 4c) showed the mean bias is -0.66, a 95% confidence interval of (− 2.44 to 1.12) %. The correlation coefficient for Hct is 0.739 (Fig. 4d), and the result is also shown in the 95% confidence interval of all data in the Bland–Altman analysis (Fig. 4e). These results show that more than 95% of the difference between the results of the device and the lab test is within this performance criterion. Table 1 also showed the comparison result of Hb levels in our device with those of the lab tests. Also, the detection limit (LOD) of the developed device was obtained from the regression curve, and the LODs were 0.75 mg/dL for PFHb, 2.14% for Hct.

**Table 1:** Comparison results of Hb levels between lab test and the proposed device.

Hemoglobin [g/dL]												
Sample number	1	2	3	4	5	6	7	8	9	10	11	12
Lab test	9.6	10.1	12	11.2	10.7	10.1	10.8	8	9.5	9.1	9.2	9.6
Proposed POCT device	9.2	9.2	11.8	11.6	11.8	9.9	9.9	9.2	10.4	10	9.1	10.3

The Hb levels of our device are derived from the Hct results.

The precision was quantified by measuring each blood sample three times repeated, and each blood sample corresponding to the value of PFHb (14.5 mg/dL, 53 mg/dL) and Hct (30%) was measured separately by our device and the reference methods. Table 2 shows the precision results, our device measured 15.13 mg/dL (for PFHb value of 14.5 mg/dL), and the standard deviation (SD) and the coefficient of variation (CV) were 1.44 mg/dL and 9.49%, respectively. For PFHb value of 53 mg/dL, the device measured 53.08 mg/dL, and SD and CV were 1.36 and 2.56, respectively. In addition, for Hct 30%, an average 29.13% were measured, and SD and CV were 1.24% and 4.26%, respectively. Therefore, from these results, the proposed device showed that it is possible to measure various blood parameters with in precision in the MCS environment.

**Table 2:** Precision results of the proposed POCT device.

		Proposed POCT device		
		Mean	SD	CV (%)
PFHb [mg/dL]	14.5	15.13	1.44	9.49
	53	53.08	1.36	2.56
Hct [%]	30	29.13	1.24	4.26

Each blood sample was measured repeatedly three times, and the SD and CV were computed.

## Discussion

We have described the device that automatically analyzes blood based on colorimetric methods, and found that the advantage is a portable blood analysis device that can be used to immediately measure in the MCS environment.

Patients supported with the MCS system are at great risk of developing hematological problems, such as hemolysis, bleeding, and thrombosis. Due to the importance of blood transfusion, there is device that continuously measure total hemoglobin in the clinical setting^29,30^. However, there is no device can simultaneously measure these diverse hematological parameters, including PFHb, which is necessary for the diagnosis of hemolysis. In addition, the method performed in the clinical setting takes a long time to obtain the results of the analysis, and a specialist has to be present who can use the analytical device. Therefore, in this study, we aimed to develop a device that could rapidly analyze the hematological parameters of patients with the MCS system by considering user convenience and the time consumed for analysis.

To measure hematological parameters in the clinical, the blood sample must be treated by chemical methods or centrifugation, and more than (50–200) µL of blood is required to analyze each lab test. We therefore embedded a small centrifuge system into the device to simplify the complicated procedure, and enable immediate analysis on site. Our device also has the advantage that it can be used without any preprocessing procedures, because it simply uses the color values of the image with separated blood. In addition, the device can measure and estimate all three hematological parameters presented in this study with only 35 µL of blood. The measurement range of PFHb is (0–100) mg/dL with a LOD of 0.75 mg/dL; Hct can be measured in the range (20–50) % with a LOD of 2.14%; and Hb can be estimated by Hct measurement. For each measured parameter (PFHb, Hct), the Bland–Altman analysis showed that ​they were within 95% confidence intervals, demonstrating the high accuracy of our device compared with lab tests. In addition, the quantitative analysis was performed by comparing the clinical lab tests with the developed device. The spectrophotometric method of measuring PFHb has a precision of SD 5.2 mg/dL and CV of 9.4% for PFHb value of 5.2 mg/dL in the range of (0.3–62.5) mg/dL. This method cannot be automated because the technician has to estimate the PFHb by measuring the absorbance at three wavelengths (380 nm, 415 nm, 450 nm)^31^. The Hb measuring device ADVIA 120 Hematology analyzer (Siemens AG, Germany) has an accuracy of SD 0.14 g/dL and CV 0.93% for Hb value of 15 g/dL in the range of (0.0–22.5) g/dL. This device has very high precision, but requires toxic chemicals and pre-processing of the sample. However, since our device separates blood cells and plasma with its own centrifuge system, and simply uses color values to measure blood parameters, it does not require toxic chemicals and pre-processing, and can measure in a short time (about 5 min). The precision analysis results of the developed device have SD 1.44 mg/dL and CV 9.49% for PFHb value of 14.5 mg/dL, and SD 1.36 mg/dL and CV 2.56% for PFHb value of 53 mg/dL. In addition, for Hb value of 10 g/dL derived from Hct 30%, the precision of SD 0.40 g/dL and CV 4.18%. Therefore, our device has the potential to replace the existing analysis method in MCS environment.

A limitation of this study is that validation was performed for a limited range of PFHb, Hct, and Hb levels. Since the condition of the animal should be kept stable during the experiment, the blood sample used for the verification of the proposed device did not deviate significantly from the normal blood range. It is reported that if PFHb levels rise above a certain hemolysis range (Mild), the risk of acute renal failure and thrombus formation increases. Therefore, we have shown high accuracy results in this mild hemolysis range of (15–60) mg/dL, which requires additional attention when monitoring patients^32^. Also, it is considered that the accuracy is more promising at the higher hemolysis range above the mild hemolysis range (~ > 60 mg/dL), because the higher the concentration of PFHb, the stronger the intensity of the color. In addition, the R value of Hct is relatively lower than the R value of PFHb. These results can be presumed to be due to the limited range of measurements, since during animal experiments, the Hct range of blood did not significantly change. However, the normal Hct level in swine is usually (35–40)%, the results meet the range of Hct < 30% and Hb < 12 g/dL as considered anemia^33,34^, and the results also show that the device has accuracy in this range. Therefore, the performance of our device could be sufficient to analyze Hct and Hb levels. In addition, in quantitative analysis, the three replicates could be slightly insufficient, but, in the MCS environment, blood cells are constantly stressed by pumps, making it difficult to obtain exactly the same value even if several samples are taken within a short time in the same animal. Therefore, we performed quantitative analysis on only three samples obtained from the same animal that completely consistent with the laboratory test results. However, since CV has less than 10% even in three replicates, it is expected that higher precision can be obtained through further studies to improve the performance of the device.

In conclusion, we propose a PFHb and Hct measuring device that can be used rapidly and intuitively in the MCS environment. The device integrates a centrifuge with a control and analysis system to reduce the time from sample collection to analysis results. In addition, since PFHb and Hct are measured based on the color information of the centrifuged blood image, the technique does not require complicated preprocessing or reagents, and the device can be easily used without professional training. Therefore, we anticipate that our device will increase the convenience of diagnosing blood parameters in patients, and also has the potential as an analytical device that is rapid, easy-to-use, and portable, which meets the needs of the MCS environment. Finally, we intend to further improve the performance for on-site diagnostic hematological analysis equipment. A possible plan is to apply colorimetric and spectrophotometry simultaneously to improve measurement resolution, and to measure more hematological parameters. We also intend to develop a network system using mobile software that enables measured results and records to be checked, even when users are not in the field.

## Methods

### Centrifuge system

The centrifuge system consists of a 12 W brushless DC motor (EC-max22, Maxon Motor, Switzerland) and a motor driver unit (ESCON 50/5 Servo Controller, Maxon Motor, Switzerland) for stable operation at high speed^35^. Chip-on-board LEDs (SY-LD1003, SMG, China) were used to give strong light intensity at low power (12 V, 100 mA), and resistors (120Ω) were used to deliver a constant current to both LEDs so that they could maintain a constant light intensity. The two LEDs were placed on both sides of the cartridge holder, so that the light intensity did not vary depending on the position of the channel cartridge. In addition, the optical diffusers suitable for the size of the LED were fabricated using acrylic plate (3 mm), so that the light was not directly transmitted to the channel cartridge and could spread uniformly in the centrifuge chamber. The camera module (Pi camera ver.2.1) is placed in the lid of the centrifuge chamber, to obtain a front view of the cartridge (Supplementary Fig. S1 and Fig. S2).

### Software process

To carry out the analysis, the user puts the customized channel cartridge into the holder, and presses the start button on the user interface to activate the entire system. To centrifuge blood, the motor rotates at about 200 × *g* (8,000 RPM) for 3 min^35^. When it slowly decelerates to 100 RPM, the stop signal is transmitted. The holder is then magnetically coupled with the housing, and held in a certain position to capture the image. When the position of the holder is fixed at the same time as the stop signal, the image of the channel captured with the camera module is transferred to the image processing algorithm. The image processing process is described in detail in Sect. 2.2, and when the analysis result is obtained, it is shown on the user interface. The user interface uses a 5-inch touch screen for easy operation. The custom software was created using Qt creator to construct a graphical user interface (GUI), and all code, including motor control and image analysis, was written using Python 3.6.1.

### Customized channel cartridge and holder design

The dimensions of the channel cartridge are 29.5 mm (L) × 24 mm (W) × 6 mm (H), and the dimensions of the channel in the cartridge are 15 mm (L) × 1.5 mm (W) × 2 mm (H). The channel cartridge was developed specifically for this system, and made of acrylic material using a laser cutting machine (MYM-1409, MYCNC, Korea). The cartridge consists of three parts, the lower, middle, and upper parts, and all parts have the same thickness of 2 mm. The hole in the upper part is the blood chamber, and after the blood is dropped into the chamber, it is covered with a black cap to prevent the blood from flowing outside (Supplementary Fig. S3). The color of the cap serves as a marker for chroma difference (△C) when analyzing images. The holes on both sides of the blood chamber are used to fix the cartridge at a given position in the holder.

The cartridge holder consists of two parts, the lower and the upper part, which parts were manufactured using a 3D printer (Stratasys F123 Series, Stratasys, Israel). The lower part of the chip holder has a magnet on the opposite side of the channel, so that the induced magnetic coupling between the housing and the holder can align the cartridge in the specified location at all times. At the bottom of the channel, the white acrylic plate (25 mm (L) × 8 mm (W) × 3 mm (H)) is inserted to emphasize the color of the centrifuged plasma. The upper part of the holder is designed so that the camera can easily acquire the channel image and the ROI (Supplementary Fig. S4). In addition, the axis of rotation of the motor is aligned with the central axis of the blood chamber in the cartridge, so that when centrifugal force is applied, all the blood can move to the connected channel.

### Calibration method for PFHb levels

Supplementary Figure S5 shows the procedure for the calibration of PFHb. First, about 50 mL of blood was collected in a conical tube, and stressed for about 1 min using a vortex stirrer to cause severe hemolysis. We used the centrifuge to obtain only the plasma of the stressed blood, and this plasma was defined as the reference solution (Original). The original solution was diluted with phosphate buffer saline (PBS pH 7.4, Gibco BRL, USA) at ratios of (1:10, 1:15, 1:20, 1:30, 1:50, 1:65, 1:85, and 1:100), to obtain samples within the appropriate range of PFHb level. The samples with more than 1,000 mg/dL of PFHb were visually very red, and the red color of the solution decreased, depending on the degree of dilution. Diluted solutions in each ratio were sent to the laboratory to obtain gold standard PFHb levels ​​that were the basis of the calibration curve. According to the guideline for the ECMO patients, the hemolysis stages are divided into four stages based on ECMO patients: Normal (< 30 mg/dL), Mild elevated ((30–50) mg/dL), Moderate elevated ((50–70) mg/dL), and Critical (> 70 mg/dL)^32,36^.

### Animal preparation and blood sample acquisition during MCS

All animal protocols were reviewed and approved by the Seoul National University Bundang Hospital Institutional Animal Care and Use Committee (IACUC No. BA1705-223/040-07), and all animal experiments were performed in accordance with relevant guidelines and regulations. The study is in compliance with ARRIVE guidelines for the in-vivo studies carried out on animals. Two female swine of 80–100 kg were fasted overnight with free access to water preoperatively. Premedication consisted of subcutaneous injection of atropine 0.05 mg/kg and intramuscular injection of xylazine 3 mg/kg and zoletil 5 mg/kg. After induction of anesthesia, swine were intubated via mouth with 7.5 mm endotracheal tube and connected to mechanical ventilator. Anesthesia was maintained with sevoflurane 1% and supplemented by oxygen 2 L/min with inspired oxygen fraction of 40% and by vecuronium bromide 0.1 mg/kg for muscle paralysis. Using the Seldinger technique, two 17-Fr cannulae were catheterized into femoral artery and opposite side femoral vein. After administration of heparin 400 unit/kg, two kinds of ECMO devices, SACCS-01 (CEBIKA, Korea) and Bioconsole-550 (Medtronic, Watford, UK), were connected to the cannulae for the MCS.

Blood samples of 10 ml were obtained via arterial line at every hour after the ECMO devices started. A 35 µL of each blood sample was used for test of the device, and the remaining blood was used for laboratory tests covering PFHb, activated clotting time (ACT), arterial blood gas analysis (ABGA), and complete blood count (CBC), including Hct and Hb. We finally sacrificed the animal with high dose potassium chloride injection by animal experiment guideline.

## Supplementary Information

Supplementary Information
