# Supplementary material for: Point-of-care testing of plasma free hemoglobin and hematocrit for mechanical circulatory support
Source: Sci Rep. 2021 Feb 15;11:3788. doi: 10.1038/s41598-021-83327-5 (PMC7884396; doi:10.1038/s41598-021-83327-5)
Supplement: Supplementary file 1 — Supplementary Information [file 41598_2021_83327_MOESM1_ESM.docx]

**Supplementary Materials**

**Point-of-care testing of plasma free hemoglobin and hematocrit for mechanical circulatory support**

**Dong Ah Shin^1^, Jung Chan Lee^2,3,*^, Heean Shin^1^,Young-Jae Cho^4^, Hee Chan Kim^2^**

^1^Interdisciplinary Program in Bioengineering, Graduate School, Seoul National University, Seoul 08826, Republic of Korea

^2^Department of Biomedical Engineering, College of Medicine and Institute of Medical and Biological Engineering, Medical Research Center,
Seoul National University, Seoul 03080, Republic of Korea

^3^Institute of BioEngineering, Bio-MAX Institute, Seoul National University, Seoul 08826, Republic of Korea

^4^ Division of Pulmonary and Critical Care Medicine, Department of Internal Medicine, Seoul National University College of Medicine, Seoul National University Bundang Hospital, Seongnam 13620, Republic of Korea

*email: [ljch@snu.ac.kr](mailto:ljch@snu.ac.kr)

**Supplementary Figures**


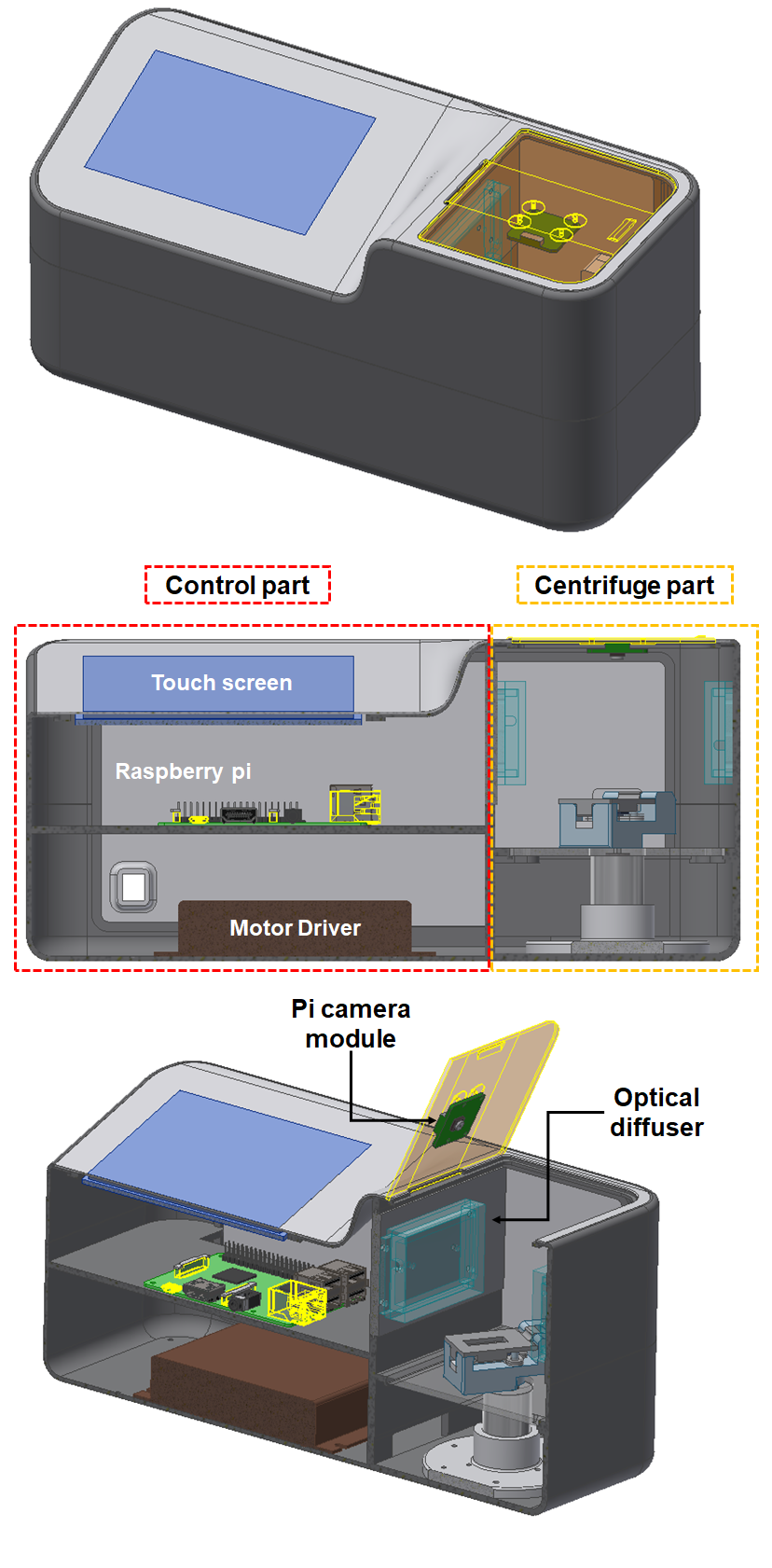


**Figure S1**. Cutaway views for our POCT device are provided for better visualization.


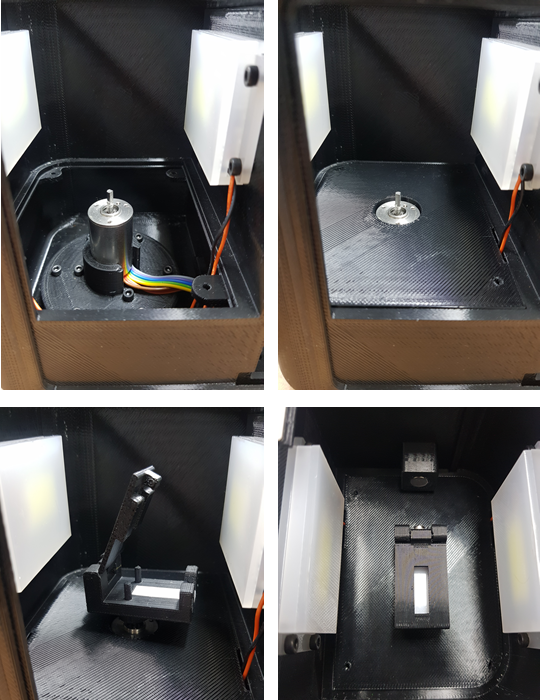


**Figure S2**. Real image of centrifuge system

**
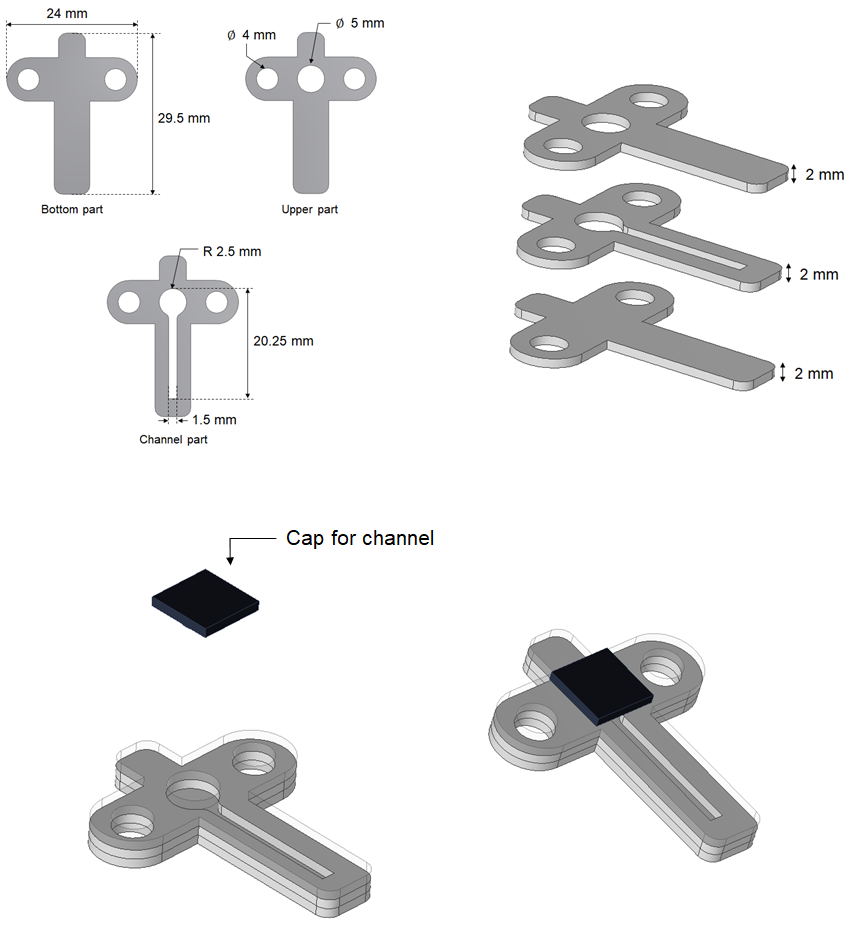
**

**Figure S3**. Dimensions of custom channel cartridges and how to make them. Each cartridge part made of acrylic was attached using acrylic adhesive. The cap of the channel was attached to the centrifuge after blood was injected.

**
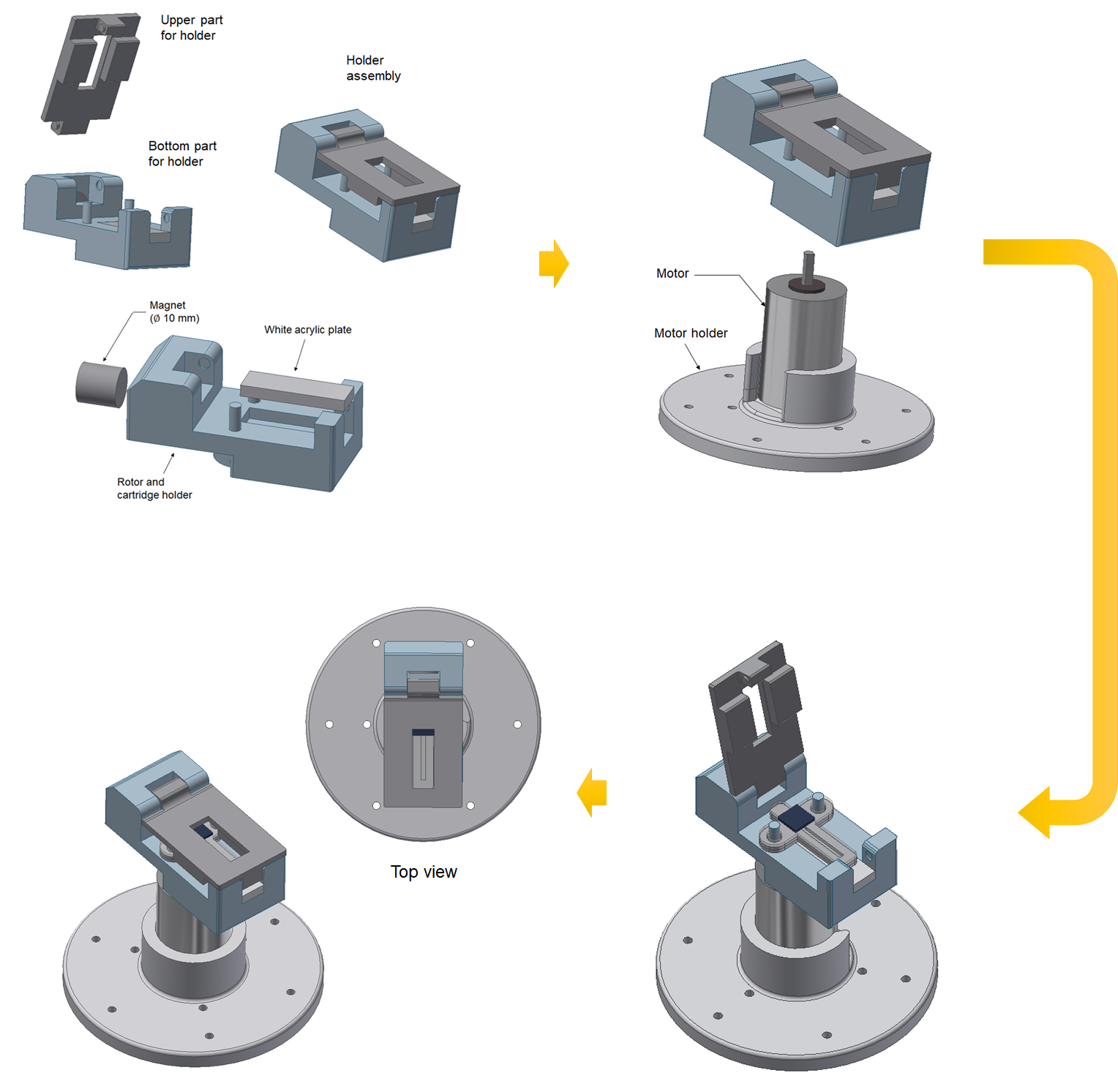
**

**Figure S4**. Centrifuge manufacturing process. The white acrylic plate was used to emphasize the color of the plasma, and the magnet was used for fixed position.

**
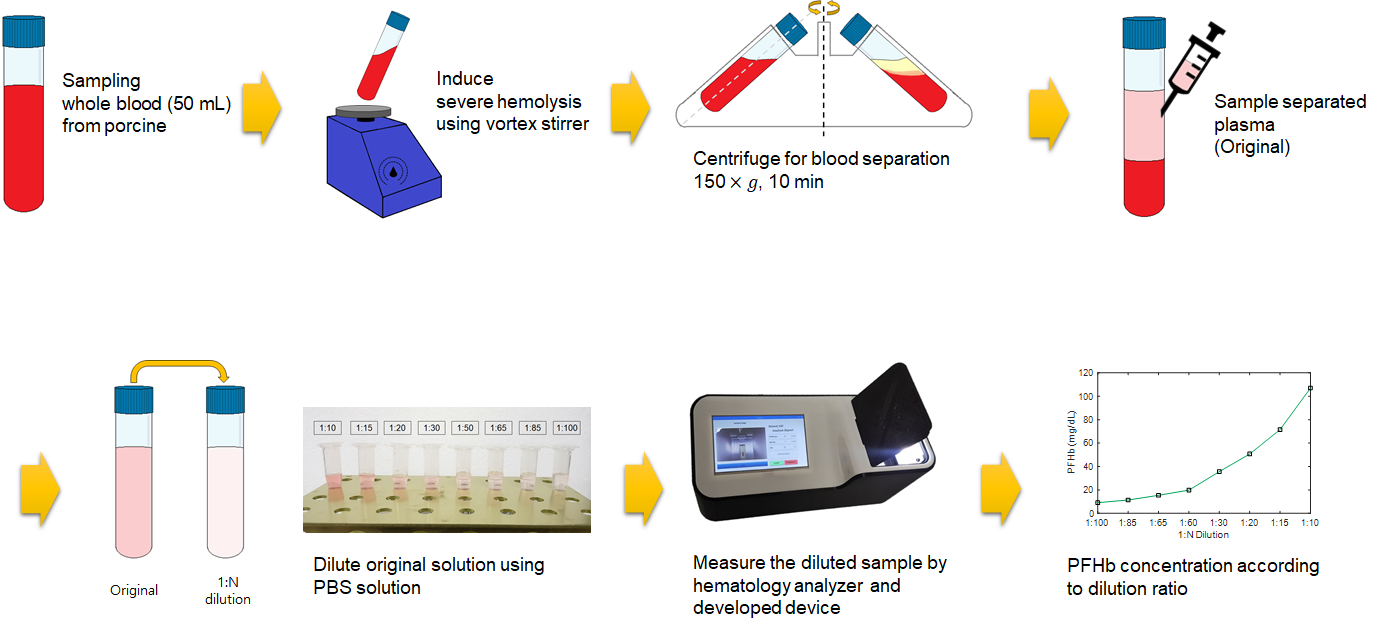
**

**Figure S5**. The calibration methods of the PFHb. Severe hemolysis was induced using a vortex stirrer, which was diluted to obtain a range of the PFHb levels ​​required in the clinic. Each diluted solution was sent to a laboratory to obtain analytical results, which were used to obtain calibration curves.
